# From Grapes to Wine: Impact of the Vinification Process on Ochratoxin A Contamination

**DOI:** 10.3390/foods12020260

**Published:** 2023-01-06

**Authors:** Laura La Placa, Dimitrios Tsitsigiannis, Marco Camardo Leggieri, Paola Battilani

**Affiliations:** 1 Department of Sustainable Crop Production, Università Cattolica del Sacro Cuore, via Emilia Parmense 84, 29122 Piacenza, Italy; 2 Department of Crop Science, School of Plant Sciences, Agricultural University of Athens, Iera Odos 75, 11855 Athens, Greece

**Keywords:** OTA, fermentation, maceration, fining agents, aging

## Abstract

Ochratoxin A (OTA) is one of the major mycotoxins, classified as “potentially carcinogenic to humans” (Group 2B) by the International Agency for Research on Cancer (IARC), and wine is one of its main sources of intake in human consumption. The main producer of this toxin is *Aspergillus carbonarius*, a fungus that contaminates grapes early in the growing season. The vinification process, as a whole, reduces the toxin content in wine compared to the grapes; however, not all vinification steps contribute equally to this reduction. During the maceration phase in red wines, toxin concentrations generally tend to increase. Based on previous studies, this review provides an overview of how each step of the vinification process influences the final OTA contamination in wine. Moreover, certain physical, chemical, and microbiological post-harvest strategies are useful in reducing OTA levels in wine. Among these, the use of fining agents, such as gelatin, egg albumin, and bentonite, must be considered. Therefore, this review describes the fate of OTA during the winemaking process, including quantitative data when available, and highlights actions able to reduce the final OTA level in wine.

## 1. Introduction

*Aspergillus* and *Penicillium* are the main fungal genera associated with ochratoxin A (OTA) production [1,2,3] This mycotoxin consists of a dihydroisocoumarin that contains chlorine associated to l-*β*-phenylalanine [1,4,5,6,7,8].

OTA is one of the most potentially dangerous fungal metabolites for human health, with nephrotoxic, hepatotoxic, teratogenic, and carcinogenic effects [2,3,9,10]. It is theorised to be the main etiological cause for human Balkan endemic nephropathy (BEN), and it has been correlated with tumours in the human urinary tract [11,12,13,14,15]. OTA was rated as a potential human carcinogen (group 2B) by the International Agency for Research on Cancer (IARC) [16], showing that there is at least some evidence of it being able to cause cancer in humans; however, this is still inconclusive. A dose of 120 ng OTA/kg body weight (bw) is the uptake that a person can tolerate in a week (TWI), according to the European Food Safety Authority (EFSA) [17]. Nowadays, the TWI established in 2006 is no longer valid. In fact, since OTA might have genotoxic and carcinogenic effects and there were doubts concerning its kidney carcinogenicity, in 2020, EFSA experts calculated a margin of exposure (MOE), which is a ratio of two factors that determines the dose at which minor, but observable, adverse effects are seen in a given population after exposure to the substance studied. MOEs above 200, determined for non-neoplastic effects, were not worrying for most consumers, except for limited groups of younger people; on the contrary, MOEs evaluated for neoplastic properties (below 10,000) indicated potential negative effects on human health [18].

Many ingredients in food and beverage products, including coffee, grapes, cereals, cocoa, nuts, spices, beer, wine, chocolate, and bread, were contaminated with OTA [1,2,9]. Within such products, cereals and wine are the first and second sources of intake, respectively [10,19,20]. Aiming to reduce public health risks, a maximum tolerable value of 2 μg/kg in must and wines was introduced by the European Commission (EC) [21] in 2006.

OTA was first found in wine in 1996 in Switzerland [22]. Since then, its presence has been reported in many countries all around the globe [3,20,23]. Somma et al. [24] summarised OTA occurrences from the Mediterranean area, South America, and Australia from 1999 to 2010, and concluded that OTA contamination around the Mediterranean was the highest. Furthermore, wines from southern areas of Europe presented higher OTA levels than those from northern European regions. This tendency was also noted comparing wines from Southern and Northern Italy. In this regard, Pietri et al. [25] affirmed that among 96 red wines vinted between 1995 and 1997, the average concentration of the toxin in northwestern, northeastern, central, and southern Italy was 11, 81, 295, and 1233 ng/L, respectively. Similarly, according to Brera et al. [26], among 1166 Italian wine samples produced between 1988 and 2004, there was a progressive increase in the toxin levels from northern to southern Italy (average concentration: 0.05 and 0.54 ng/mL, respectively). These data suggest that climate has a strong impact on OTA occurrence. 

The incidence of the toxin in wine is a topic under continuous study. Among the most recent reports, Silva et al. [27] examined 100 wine samples, produced between 1984 and 2017, available on the Portuguese market. They detected OTA in five of them made from 2015 onwards. Another recent study, which examined 113 bottled wines (vintages 2011–2016), reported that 52% of the sampled wines possessed measurable levels of OTA (>0.02 μg/L), and red wines had higher residues of OTA than white and rosé ones (64, 43, and 36%, respectively). Moreover, differences were found among the years, with 2014 and 2015 being the most problematic harvests, reaching levels of contamination of 71 and 87%, respectively [28].

In grapes, the main source of contamination of OTA is *Aspergillus carbonarius* [2,11], while other species of black aspergilli produce the toxin in lower amounts [24,29]. Notably, Battilani and Silva [11] showed that 70–100% of *A. carbonarius* strains and only 2–20% of the *A. niger* aggregate produced the toxin in vitro. Similarly, Kizis et al. [30], who studied the ochratoxigenic potential of black aspergilli from grapes, demonstrated that 98% of *A. carbonarius* isolates and only less than 2% of *A. niger* aggregate produced OTA in vitro.

*Aspergillus* species overwinter in vineyards, and the main origins of inoculum are soil and vine debris [31]. Damaged berries due to biotic and abiotic factors, including insect or fungal attacks, rainfall, and hail, favour the penetration of the fungus into grape skin [32]. If there are no damages, the outer skin might be contaminated with spores, but they do not generate observable signs [31]. Confirming this, Jiang et al. [33] observed no symptoms or OTA production for surface-inoculated undamaged grape berries. Thus, damages to berries are fundamental to OTA synthesis in grapes according to some authors [34], but it was reported that musts obtained from bunches that do not present visible symptoms might also be contaminated; however, generally, OTA is detected in greater amounts in grapes with observable signs of black aspergilli contamination [35]. Instead, grape skins can carry OTA in wine [34,35]. Even though infections can start at the berry setting, rots develop more rapidly with berry maturity, when berries have thinner skin and contain higher levels of sugar, which favour *Aspergillus* spp. colonization and development [2]. Consequently, OTA production is abundant in the interval between early veraison and harvesting [36].

Therefore, the fungal development on berries during the growing season is the main cause for the presence of OTA in wine and the subsequent mycotoxin production; however, the toxin remains during the vinification process, being present in the final product [37]. Therefore, this study aimed to review how each step of the winemaking procedure affects the final OTA contamination in wine, taking into consideration the most noticeable recent studies on the subject. The post-harvest strategies that are available for the detoxification of this mycotoxin are also discussed.

## 2. OTA in the Winemaking Process

### 2.1. The Winemaking Process

Winemaking, or the vinification process, i.e., the production of wine, involves several steps which can differ based on wine type. However, the essential operations in winemaking are substantially the same.

Following harvest and transport to the vinery, bunches are pressed to obtain must. In red wines, as opposed to white ones, it is desired to obtain colour and flavour. Therefore, in their production, maceration is a fundamental step, while it is excluded in the production of white wines [11]. Rosé wines are an intermediate type of wine produced with red grapes, but commonly obtained from a shorter contact with skins than for red wine production.

Subsequently, during alcoholic fermentation, grape sugars are converted into ethanol (C_2_H_6_O) and carbon dioxide (CO_2_) by yeasts [8]; this passage takes from 10 to 20 days and demands constant observation to obtain high-quality wines. 

Red wines, after the solid components have been separated from the liquid through drawing-off or racking, sometimes go through malolactic fermentation; lactic acid bacteria (LAB) convert malic acid (C_4_H_6_O_5_) into lactic acid (C_3_H_6_O_3_) and CO_2_. 

At this point, a storage period in tanks may follow. Afterwards, clarification and stabilisation processes are usually performed to remove suspended solids [2]. They may involve filtration, centrifugation, and fining, i.e., the process by which a fining agent is added to the wine to create a bond with the suspended particles to help their precipitation.

The winemaking process’s final stage involves the wine’s ageing and bottling. 

### 2.2. Fate of OTA during Winemaking

The whole winemaking procedure decreases OTA content in wine. According to Leong et al. [31], an OTA reduction of above 80% occurs during vinification, regardless of the starting OTA levels in grape, thanks to the adsorption of solids contained in the must.

In most cases, a higher toxin content is observed in red wines compared to white ones, which does not solely depend on a greater contamination at harvest, but also on the vinification process, and foremost during the maceration phase. In fact, this fundamental step required in the production of red wines results in the greatest release of toxins in wine [20,24,38,39,40,41,42]. Several studies agree on the fact that red wines are generally more contaminated [11,26,43,44,45,46], with few exceptions, among which are Stefanaki et al. [47] and Lopez de Cerain et al. [48], who found little difference between white and red wines.

In a recent study, Freire et al. [10] analysed wines produced in the region of the Vale Submédio São Francisco (Brazil) from inoculated grapes (10^4^ conidia/g), and detected OTA reductions of 91, 92, and 88%, in white, rosé, and red samples, respectively, between grapes and wine. 

Nevertheless, it is important to better understand how each operation during winemaking modifies the OTA content. In the first step of crushing, when grapes containing OTA are broken to extract the juice, the toxin passes into the juice [40]. Abrunhosa et al. [49] studied artificially inoculated red and white grapes (cv. Vinhão and Loureiro; initial OTA content of 0.43–7.48 µg/kg) and reported that, after crushing, around 41% of the toxin remains in the pomaces, while the rest moves into the juice. The maceration process causes a growth in OTA levels in red wines, as the toxin present in the grape skin is solubilised and released into the must [2]. In winemaking trials conducted in Puglia, Italy, in three different vintages (2001–2003), with the grape varieties Negroamaro and Primitivo, that were naturally infected, Grazioli et al. [40] reported a 45% OTA increase after crushing and the following maceration. Dachery et al. [50] focused on Cabernet Sauvignon harvested in 2014 in Serra Gaúcha, Brazil and observed a sixfold increase in toxin concentration after the maceration phase. Nevertheless, during the following alcoholic and malolactic fermentations, OTA significantly decreased. Several studies have noted the reduction in OTA concentrations during the alcoholic fermentation. Abrunhosa et al. [49] proved that wine contains 32% of the starting concentration on the grape while, during microvinification experiments conducted in the region of Takelsa, Tunisia, Lasram et al. [51] observed a decrease in OTA levels of about 41% from grape to red wine after this step. Similarly, Grazioli et al. [40] reported a 30% OTA decrease after alcoholic fermentation and racking. 

According to Blesa et al. [38], OTA synthesis is inhibited by ethanol concentration and anaerobic conditions, which hinder fungal growth during fermentation. Similarly, Jiang et al. [3], who used five grape cultivars in Shaanxi province, China, to produce dry red wine (12% alcohol), noted that the growth and OTA production of *A. carbonarius* were significantly lowered with an ethanol concentration of 2–4%. In fact, ethanol possesses strong antimicrobial properties, so it is commonly used for improving food preservation [52] and represents an important factor in reducing OTA synthesis in wine. Furthermore, according to the authors, the addition of sulphur dioxide (SO_2_) hindered the development of the fungus and its OTA biosynthesis, to the point of completely nullifying it at 500 mg/L; on the other hand, reducing sugar concentration did not significantly affect contamination and OTA synthesis. 

The reduction in OTA from must during the fermentation process essentially happens through adsorption to binding solids such as grape skins [52]. Corroborating this, Esti et al. [53] observed the effect of alcoholic fermentation on OTA levels in must fortified with OTA, both with and without grape skins. They proved that the reduction in OTA levels was significantly (5–12%) higher when the skins were present. Yeast cell walls, mainly composed of mannoproteins, contribute to further adsorb the toxin and reduce its concentration [51]. Therefore, likely due to their different cell wall composition, the yeast species and strains involved in the process do not have the same ability to reduce toxin levels. In this regard, Caridi et al. [54] found that 20 strains of *S. cerevisiae* reduced the toxin by 68% in naturally contaminated wine during alcoholic fermentation, from an initial concentration of 1.58 ng/mL to 0.14–0.95 ng/mL; furthermore, OTA levels in wines produced from must fortified with OTA were reduced by about 78%, from 7.63 ng/mL to 1.27–2.45 ng/mL. This wide range of values proved that a considerable reduction in the original OTA content was achieved with all the tested strains, though they possess different toxin-reducing capabilities. Similar results were also obtained by Meca et al. [55], who studied the process of alcoholic fermentation of Moscato wine using 16 strains of *S. cerevisiae* and found that detoxifying capabilities vary depending on the strain, from 32% to 50%.

Furthermore, some studies reported that this step of the winemaking process had a greater effect in decreasing OTA levels in red wines than white wines, probably due to a chemical bond between anthocyanins and OTA in the red wine [51]. Indeed, Cecchini et al. [56] analysed wines produced from one white must and two red musts obtained by adding anthocyanin extract in two different concentrations to the original white must. They observed OTA reductions in all wines, though they were the highest in red wines. Csutorás et al. [57] focused on OTA concentration during an alcoholic fermentation process which took place over 90 days in macro-scale trials with musts fortified with OTA at different levels. They demonstrated that OTA reduction in red, rosé, and white wines was 89%, 85%, and 75%, respectively. Moreover Cecchini et al. [5] stated that the OTA reduction was 47–52% in white wine (from Trebbiano Toscano and Malvasia del Lazio grapes), and 53–70% in red wine (from Primitivo grapes), while, according to Dachery et al. [50], OTA reduction in red and white wines was 54% and 35%, respectively.

In red wine production, the malolactic fermentation that follows the alcoholic one can also be related to OTA decontamination. The removal of OTA during this step is not probably due to its degradation by the LAB, but to its adsorption on the cell wall or on other suspended solid particles [51].

*Oenococcus oeni* is the principle bacterial species utilised in malolactic fermentation, with certain strains capable of removing OTA [2]. Mateo et al. [58] studied the ability of ten strains of *O. oeni* to eliminate OTA from culture media exposed to varying conditions after 14 days of incubation; they found reductions of 36–63%, depending on both the strain and the starting OTA contamination. Grazioli et al. [40] observed a 56% OTA decrease in two naturally contaminated Italian red grape varieties during malolactic fermentation and racking. On the contrary, Lasram et al. [51] reported that this step had little effect on OTA content. Similarly, Abrunhosa et al. [49] affirmed that reductions of 3% of OTA concentrations in wine may occur during this stage. Variation in the technical procedures, or most probably the strains involved in the fermentation, may explain these differing data regarding the effect of malolactic fermentation [51].

Another step that contributes to OTA decrease is clarification. According to Leong et al. [59], the bond between OTA and sediment is so strong that it cannot be broken even by a centrifuge. Indeed, centrifugation results in the compaction of precipitated solids, and can be helpful in improving the recovery of juice or wine from the lees with no increase in OTA contamination. The authors also highlighted that fining greatly impacted OTA. Among the main adsorbents stated as being effective in removing OTA from contaminated wines are activated carbon, bentonite, gelatin, egg albumin, potassium caseinate, and natural polymers, such as chitin and chitosan [2]. The effect of these products will be treated separately in Section 3.3. 

As regards filtration, Gambuti et al. [34] studied microfiltration techniques using 0.45 µm membranes, and observed a reduction in OTA levels of 80%, likely correlated to the fact that OTA binds to the macromolecules which are retained by the membrane. The authors used grapes cultivated in the Taburno DOC area (Italy). Durguti et al. [60] analysed 54 samples of newly fermented wine (grape production 2013) and concluded that proper clarification and filtration are useful in reducing, or possibly eliminating, OTA.

The storage period before or after clarification might also contribute to OTA decrease. Lasram et al. [51] proved that a five-month storage period and subsequent draining resulted in a considerable OTA reduction of about 55%. The cause of this decrease is correlated to the precipitation of solid compounds to which the toxin binds. In a small-scale vinification trial conducted in 2004, in which Semillon and Shiraz grapes were inoculated with *A. carbonarius* (OTA concentration in the crushed grapes: 184 and 58 µg/kg, respectively), Leong et al. [59] found that storing both the white and the red wine for 10–14 months achieved a reduction in OTA concentrations of 22% and 29%, respectively. The highest OTA reduction that occurs during the storage of red wine compared to white wine is likely linked to red wines having a more complex matrix, able to bind the toxin better.

Garcia-Moruno et al. [61], who analysed a 2001 red Italian wine, found that a storage period of 55 and 80 days resulted in 8% and 10% OTA reductions, respectively. As regards the wine bottle ageing, according to Anli et al. [62], an OTA decrease in red wines occurred after storing the wine for 12 and 18 months, of 12% and 18%, respectively; furthermore, the 6-month storage had no effect, and the 24-month storage was more effective than 18 months. Similarly, Grazioli et al. [40] noted a 17% OTA decrease in Negroamaro wine after 12 months of storage in bottles and no appreciable effect with shorter ageing.

### 2.3. Sweet Wines

Separate observations are necessary for sweet wines, wines with a residual sugar content of at least 45 g/L [63], and dessert wines. High sugar content can be obtained through various techniques. These mainly include adding exogenous sugars before or at the end of the fermentation, adding alcohol to prevent the fermentation of all the sugar (a process called “fortification”), and removing water to concentrate the sugar [64].

Burdaspal and Legarda [65] found OTA in 281 of 290 sweet wines (97%) vinted in 2002–2005 in Spain and other nations in concentrations of 0.01–4.63 µg/L. Globally, average and median concentrations were observed to be 0.50 µg/L and 0.14 µg/L, respectively. According to Hocking et al. [66], only 23% of Australian dessert wines tested positive for OTA, with 0.50 μg/L being the highest concentration detected. This contrasts with results from a Greek study, where Soufleros et al. [44] tested seven sweet wines (three red and four white), six of which were found to contain the toxin (86%) at a mean concentration of 0.94 ng/mL. Labrinea et al. [67] also observed that 81% of the 27 tested Greek dessert wines produced during 1999–2006 were contaminated, with 22% having more than 1.0 µg/L of OTA. In studies conducted on wines from Italy, Pietri et al. [25] analysed 15 white dessert wines chiefly vinted between 1995 and 1997, and found OTA in 60% of them, with a median concentration of 8 ng/L and a mean of 736 ng/L. Similarly, Brera et al. [26] found 64% positive samples in 28 dessert wines analyzed, with a median concentration of 0.05 ng/mL and an average concentration of 0.26 ng/mL, while Di Stefano et al. [68] analyzed 30 samples of Sicilian sweet wines and 97% of them tested positive for OTA (mean of 0.25 μg/L). However, none contained OTA in concentrations higher than the permitted limit.

Some papers compare OTA content found in sweet and dry wines. Among them, Zimmerli and Dick [22] studied dessert wines (Malaya, Marsala) and found a median of 337 pg/mL. The value was much greater than the levels found in white, rosé, and red table wine (<3 pg/mL, 19 pg/mL and 13 pg/mL, respectively). Similarly, Stefanaki et al. [47] analyzed dessert wines collected in several parts of Greece between 1995 and 1999 and found a median of 0.33 μg OTA/L, which was higher than what was found in dry wine samples (0.07 μg/L). Thus, sweet wines generally retain greater OTA concentrations than dry ones, which correlates with different production techniques. For example, producing several sweet Mediterranean wines through dehydration may increase fungal development and OTA formation [69]. Similarly, in fortified wines, the addition of exogenous alcohol might originate higher OTA contamination [2]. Indeed, Valero et al. [70] performed a thorough examination regarding OTA occurrence in 121 special wines made using a range of vinting processes. The wine groups with the highest OTA concentration and incidence (>90%) were produced with a must that had been inoculated before fermentation and from grapes that had been dried naturally under the sun, which presented an average OTA concentration of 4.48 μg/L and 2.77 μg/L, respectively.

## 3. OTA Removal during Winemaking

Several strategies are available to lower OTA concentrations in wine. These can be categorized as physical, microbiological, and chemical approaches, while the Commission Regulation (EC) N 1881/2006 states that dilution with non-contaminated food products is not allowed [21].

### 3.1. Physical Methods

Reducing the time between harvest and crushing is a successful measure to reduce the risk of OTA contamination of unprocessed grapes [2] since it is important not to leave the harvested product at temperatures and water activity (a_w_) regimes that allow toxin biosynthesis. Indeed, *A. niger* aggregate strains mainly produced OTA at 20–25 °C, whereas *A. carbonarius* performed the best at 15 or 20 °C [71]; moreover, OTA synthesis is also favored by high levels of a_w_ [36]. These conditions can commonly be faced by harvested grapes if not rapidly processed. Therefore, refrigerating the grapes in case of delays in crushing could be an interesting solution to keep OTA production to a minimum [11].

As regards thermal treatments, Gambuti et al. [34] stated that filtration through a 10 μm membrane and heating on hot plates at 55 °C did not produce meaningful reductions in toxin levels.

An interesting method for OTA decontamination was proposed by Solfrizzo et al. [72]. They observed that up to 50–65% of the toxin might be eliminated through the repassage of musts or wines (spiked at 5 μg/kg OTA) over uncontaminated grape pomaces. Pomaces possess a strong affinity for the toxin and have been demonstrated to be still remarkably effective at removing the toxin, even after being reused four times. Thus, this method is useful but requires an early identification of the contaminated grapes, which must be processed separately from uncontaminated ones. Moreover, it is important to use pomaces from the same grape variety to avoid modification of wine quality parameters, such as its color intensity and its phenolic content. After eight years, Jiménez-Martínez et al. [73] found that grape pomaces (13 mg/mL), purified with either ethanol or acetone, removed 57% and 54% of OTA, respectively, in Monastrell red wine contaminated with the toxin at 0.5 µg/L. These data confirmed that OTA occurrence could be lowered by purified grape pomaces, which are, therefore, a good alternative for OTA decontamination.

### 3.2. Microbiological Methods

Different microorganisms can degrade the toxin. Bejaoui et al. [74] examined 40 isolates of *A. carbonarius*, *A. niger* aggregate, and *A. japonicus* to assess their OTA degradation capabilities within a medium of synthetic grape juice with the toxin at 2 mg/L. They all lowered OTA levels by at least 30%, and 77% of them were able to reduce the toxin by more than 80%. Moreover, *A. niger* performed the best, followed by *A. japonicus* and *A. carbonarius*. Bejaoui et al. [75] confirmed the beneficial effects of black *Aspergillus* isolates in synthetic and natural grape juices due to OTA adsorption on conidia, but also for degradation to ochratoxin α (OTα). Moreover, unlike the previous study, the authors found that among the three species, *A. carbonarius* detoxified grape juice most effectively since its conidia had a larger surface to bind the toxin compared to those of the other two species. In fact, in a synthetic grape juice with starting OTA levels of 2 mg/L, with a concentration of 10^7^ conidia/mL, 10%, 28%, and 45% OTA decreases were observed for *A. niger*, *A. japonicus*, and *A. carbonarius*, respectively. In a red grape juice spiked with the same amount of 2 mg/L, OTA removal by living conidia at 10^7^/mL was around 30% for *A. japonicus* and *A. niger*, and 55% for *A. carbonarius*, while with heat-treated conidia at the same concentration, it was also better, about 42%, 48%, and 67% respectively. These are interesting scientific results, but the addition of these microorganism is not recommended as a winemaking practice.

During alcoholic and malolactic fermentation, OTA levels decrease, as previously reported. Several yeasts have been reported as detoxifying agents. This is attributable both to an adsorption phenomenon to their cell walls and to enzymatic degradation. In the phenomenon of adsorption, the toxin links to yeast cell wall elements, and this process is not dependent on cell viability. Consequently, there seems to be an obvious benefit in detoxification through microbial adsorption, as it may be carried out using autoclaved yeast cells. In this way, the substrate components are not consumed, and secondary metabolites are not freed, preventing the wine’s characteristics from changing [2]. Indeed, Farbo et al. [76] proposed calcium alginate spheres with yeast cells that had been autoclaved as a good alternative to eliminate residues of the toxin from wine. The authors found that adding autoclaved yeast cells, either free or entrapped in calcium alginate, to commercial grape juice spiked with OTA led to an OTA reduction >80% after 48 h of incubation. Various inactivated yeasts, or their cell wall preparations, and strains of *S. cerevisiae*, *S. bayanus*, *Candida intermedia*, *C. fiedrichii*, and *Lachancea thermotolerans* have proven their capabilities to adsorb the toxin in wine. Fiori et al. [77] evaluated two non-fermenting (*Cyberlindnera jadinii* 273 and *C. friedrichii* 778) and two low-fermenting (*C. intermedia* 235 and *L. thermotolerans* 751) yeast strains against *A. carbonarius*, both in vivo and in in vitro trials, for their ability to remove OTA from grape juice. They found that certain strains could reduce *A. carbonarius* occurrence and/or greatly adsorbed artificially spiked OTA from the juice. Moreover, autoclave treatment of yeast cells showed that all four strains had higher OTA adsorption abilities, in agreement with previous reports. 

Bejaoui et al. [78] compared six viable and dead (heat- and acid-treated) *Saccharomyces* strains (five *S. cerevisiae* and one *S. bayanus*) to a commercially available yeast walls additive for their ability to remove OTA in both natural and synthetic grape juices. All the strains reduced OTA more effectively than the commercial yeast walls additive, and dead strains were more effective than viable ones. In fact, according to the authors, heat treating yeast cells can result in a noticeable improvement in their OTA-adsorption properties. Different conclusions were achieved by Petruzzi et al. [79]; they examined the OTA adsorption capabilities of two strains *of S. cerevisiae* (the wild strain W13 and the commercial isolate BM45), after having been heat inactivated, as well as a yeast cell wall preparation. From an initial OTA content of 2 µg/L, they concluded that yeast cell walls obtained better results (50% vs. 43% OTA decrease, respectively) compared to heat-inactivated cells. However, yeast cells might impact wine color. 

### 3.3. Chemical Methods

Some fining agents used in the clarification process actively reduce OTA levels in wine. Two important products often applied during vinification are gelatin and egg albumin. These are positively charged proteins at the pH of the wine, and they may be capable of interacting with the toxin through hydrogen bonds [80]. Many authors have studied the effect of these two substances on OTA in wine, but they reached conclusions that are sometimes contradictory. All data regarding OTA removal with fining agents are reported in Table 1.

Lasram et al. [51] reported that clarification with gelatin favored the elimination of about 58% of OTA from red wine obtained from naturally contaminated grapes in the winery (from 0.662 µg/L to 0.279 µg/L), and, according to Castellari et al. [81], this fining agent was able to remove 30% and 17% of the toxin from two red wines with starting values of 3.78 and 1.50 ng OTA/mL, respectively. Zhang et al. [90] showed that 0.1% gelatin eliminated 28% of OTA in Cabernet Sauvignon red wine, and Quintela et al. [80] stated that the use of gelatin at 16 g/hL resulted in the highest OTA reduction (up to 39%) when compared to other fining agents that they added to red wines spiked with OTA at 2.5 µg/L. On the other hand, according to the authors, egg albumin only achieved an OTA reduction of up to 16%, affecting the color intensity (CI, i.e., the saturation of a color) value and CIELab parameters (three values, L*, a*, and b*, that the International Commission on Illumination–Commission Internationale de l’Eclairage, CIE, has fixed, and which are used to express the colour space; L* stands for lightness, a* for red/green value, b* for blue/yellow value). On the contrary, Anli et al. [62] found that isinglass (10 g/hL) and egg albumin (10 g/hL) produced a higher decrease in OTA (21% and 20%, respectively), compared to gelatin (10 g/hL; 14% OTA decrease) in naturally contaminated red wines with 0.51 µg OTA/L. According to Fernandes et al. [52], the most efficient OTA removal was in red wines treated with egg albumin (0.1 g/L) and gelatin (0.1 mL/L) (22–34% and 8–21% reduction, respectively, depending on the initial OTA amount: 0.73–0.56 mg/L). Sun et al. [82] stated that treatment with egg albumin (0.2 mg/mL, 48 h) led to a greater OTA reduction (94%) than gelatin (0.2 mg/mL; 80% of reduction) in Cabernet Sauvignon wine with a 5 ng/mL starting OTA concentration. One year later, Jiménez-Martínez et al. [73] obtained different results in a red wine artificially contaminated with 0.5 µg OTA/L. They tested a gelatin with a high degree of hydrolysis (0.15 g/L), a gelatin with a low degree of hydrolysis (0.10 g/L), and an egg albumin powder (0.15 g/L) and found that the two gelatins were not effective in reducing the toxin (0 and 3% reduction, respectively), and the use of egg albumin only decreased the OTA content by 9%. 

Another thoroughly tested fining agent is bentonite, a volcanic clay belonging to the sheet-structured montmorillonite group. Salaha et al. [83] stated that a 34–40% OTA reduction was observed with 0.8 g/L bentonite, starting with 3.15 ng OTA/mL, while in the same year, Fernandes et al. [52] found that 0.5 g/L bentonite removed 7–17% of the toxin in a white wine with an initial OTA amount of 1.2–2.0 mg/L, and Jiménez-Martínez et al. [73] found that bentonite (0.75 g/L) produced a decrease in OTA levels of 17%. According to Leong et al. [59], bentonite in a Semillon wine with 8 μg OTA/kg removed 67% of the toxin, but they used a higher dosage of the agent (2.5 g/L). Sun et al. [82] found that bentonite had a poor OTA removal rate (10%), which probably also derives from using lower doses (0.1, 0.15, and 0.2 mg/mL) of the fining agent. Comparable conclusions were achieved by Visconti et al. [42]. Moreover, Anli et al. [62] reported a 20% OTA decrease in white wines (mean OTA content before treatment: 0.49 µg/L) with bentonite at 50 g/hL, and Zhang et al. [90] showed that 1% of bentonite removed 23% of OTA in the red wine previously mentioned. However, Quintela et al. [80] studied the effect of activated sodium bentonite at 10, 20, 40, and 60 g/hL in red wines spiked with 2.5 µg OTA/L and noted that at 10 g/hL, there was a 22% OTA decrease and at 60 g/hL there was a 33% decrease. The authors also affirmed that the treatment affected some wine-quality parameters, particularly the CI value. On the contrary, Castellari et al. [81] and Var et al. [45] reported that bentonite and sodium bentonite are not very effective in reducing the toxin.

Kurtbay et al. [87] used natural, nonylammonium, dodecyl ammonium, and KSF-montmorillonite bentonites and noticed that in red wines with an initial OTA content of 2.71 ng/mL, KSF-montmorillonite bentonite performed the best. 

Regarding carbon and activated carbon (AC), several studies reported their high affinity with OTA. Var et al. [45] tested AC in white wine at different concentrations (20, 40, and 100 g/hL), incubation times (0, 4, and 24 h), and OTA concentrations (5, 10, and 20 ng/mL), and found that OTA adsorption was strongly influenced by both AC and toxin concentrations, and incubation time was also relevant. Up to 100% and 98% of OTA, respectively, was removed by adding 1 mg/mL AC to phosphate-buffered saline (PBS) and white wine samples containing 5 ng OTA/mL. Using the same dose for the treatment (1 g/L), Cosme et al. [85] reported complete OTA reduction in a spiked (10 μg/L) commercial white wine, with an enhancement of the chromatic properties. The authors also reported that only the AC with more mesopores entirely removed the toxin from red wines, probably because anthocyanins are attracted by mesopores as well. Castellari et al. [81] obtained the best results in reducing OTA levels with AC. A study by Anli et al. [62] came to different conclusions, stating that AC was no more effective at removing OTA than the other fining agents (17% OTA decrease from white wines, starting with an average content before treatment of 0.49 µg OTA/L). Visconti et al. [42] found that carbon, or commercial preparations containing it, achieved the best results. However, the authors observed that these two fining agents also decreased the polyphenol content of wines. Gambuti et al. [34] found that only oenological carbon reduced OTA concentrations. In fact, they found that oenological decolorizing carbon (30 g/hL) managed to remove 73% of OTA (from 4.38 to 1.19 g/hL). Moreover, unlike the previous study, the treatment did not seem to have affected the polyphenol content and color of red wine, even though the authors noted a reduction in fundamental sensory molecules, such as 3-methylbutyl acetate, ethyl hexanoate, ethyl octanoate, and geraniol. Finally, Olivares-Marín et al. [84] examined the ability of AC prepared from cherry stones (CS) by activation with H_3_PO_4_, ZnCl_2_, or KOH to eliminate the toxin from two naturally contaminated Italian red wines (Shiraz wine 2005 and Sangiovese wine 2006) with initial amounts of 7.38 and 2.36 µg OTA/L, respectively. The authors found that AC prepared by KOH activation at 900 °C (3:1 KOH:CS) performed the best since it reduced more than 50% of the toxin, while it only slightly affected some organoleptic parameters including the total polyphenolic index, color intensity, and hue.

Anli et al. [62] tested the effect of the mixed application of certain fining agents. They reported that gelatin–bentonite and casein–gelatin–bentonite resulted in an OTA decrease of 20% and 21% in white wines with an average content before treatment of 0.49 µg OTA/L.

Nevertheless, specific fining agents containing products derived from eggs, fish, and dairy could cause allergic reactions in susceptible wine consumers. Therefore, fining agents that do not use products containing allergens have been developed.

Quintela et al. [80] assessed the OTA reduction capacities of a complex consisting of polivinilpolipirrolidone (PVPP), plant protein, and amorphous silica (in place of casein and potassium caseinate); as reported previously, they used red wine spiked with 2.5 µg OTA/L. Such agents reduced OTA levels by up to 40% at a dosage normally used in winemaking. Nevertheless, the complex affected some wine quality parameters. 

Nowadays, increased attention has been noted in the use of polymeric substances such as PVPP. Indeed, in a recent study, Carrasco-Sanchez et al. [89] found that 5 mg/mL of poly(acrylamide-co-ethylene glycol-dimethacrylate) (PA-EGDMA) removed 68% of OTA from a commercial red wine produced in Chile (Cabernet Sauvignon, 2013), with the highest reduction in phenolic compounds of 13%.

Other alternative adsorbents used for OTA removal in wine are non-toxic, biodegradable polymers such as chitin, chitosan, and their derivatives. Quintela et al. [80] stated that 500 g/hL of chitin in red wine removed 29% of the toxin without significantly altering the organoleptic characteristics of the product, whereas chitosan (500 g/hL) was able to remove 67%, but, at the same time, it strongly affected the CI value and the CIELab parameters. Bornet and Teissedre [86] stated that chitosan (5 g/L) and chitin (5 g/L) were able to reduce OTA by 83 and 67%, respectively, in red wine and by 53% in white wine, while, according to Zhang et al. [90], chitosan (0.1%) removed 25% of OTA in the Cabernet Sauvignon red wine previously cited. Kurtbay et al. [87] found that 1000 g/hL of chitosan could remove the toxin in red wine. However, they used a greater dosage than what is suggested during vinification procedures, and polyphenols and anthocyanins were greatly affected.

Savino et al. [88] found that bits of oak wood, widely used in countries outside the European Community to improve color stability and taste complexity and to provide characteristic aromas, could be employed effectively to lower OTA levels in wines containing the toxin. The type of wood (chips or powder), amount used, time of contact, and wine composition influenced the results. The contact of a Sangiovese wine spiked with 4 μg OTA/L with 8 g/L powder performed the best, as it reduced up to 76% of the toxin. Contact times over 15 days did not change the effectiveness of the treatment and perhaps even produced the opposite effect. In Lambrusco and Nero di Troia wines (initial OTA concentration of 1.01 and 2.86 µg/L, respectively), after 8 days of contact with 8 g/L chips, the percentages of OTA reduction were 51% and 50%, respectively. 

The results, at times contradictory, that the various studies obtained may partially be connected to the concentrations of fining agents, the chemical composition of wine, the starting toxin level, and the interference of other wine compounds, as many mechanisms intervene in the process [81]. In a red wine spiked with 1–10 μg OTA/L, Appell and Jackson [91] found that the best activity of a β-cyclodextrin-polyurethane polymer to reduce the toxin was when the initial OTA levels were between 2.5 and 10 μg/L. The authors suggested that there was a competition between OTA and the other wine constituents of similar molecular size for the binding sites of the polymer and that, especially at lower OTA levels, the binding sites could be occupied by the other wine constituents. Similarly, Loffredo et al. [92] stated that the other elements of wine, such as polyphenols and anthocyanins, affected OTA removal. In the aforementioned paper, Castellari et al. [81] affirmed that AC was the most efficient adsorbent, since it interferes to a limited extent with the other wine compounds and greatly reduces the toxin without affecting the total polyphenol content of red wine. According to the authors, the removal of OTA is strongly influenced by wine polyphenols. Var et al. [45] agreed that some elements of wine interfere with the OTA reduction by fining agents, as they observed that AC was more effective in reducing the toxin in white wine than in PBS. Similarly, as already reported, Savino et al. [88] concluded that the lower percentage of OTA reduction in Lambrusco and Nero di Troia wines could be explained by the interference of wine anthocyanins in the adsorption of OTA by wood chips; in fact, they detected higher total and monomer anthocyanins in these two wines compared to Sangiovese.

Furthermore, as shown by certain studies, it must always be kept in mind that these fining agents can alter some organoleptic characteristics of wine, even when they are employed within the recommended quantity. Thus, when using fining agents, it is important not to change the flavors and colors of the wine [2]. Indeed, Abrunhosa et al. [49] confirmed that chemical fining agents might be useful in reducing the toxin. At the same time, they may affect other organoleptic properties of the final product.

## 4. Modified Ochratoxins

The decrease in OTA concentrations during the vinification process is also linked to the degradation or transformation of the toxin [10]. These “modified ochratoxins” are OTA metabolites that usually remain undiscovered when testing for the parent mycotoxin. Despite their lack of regulation in wine, their presence poses potential threats to human health.

The most relevant OTA derivates present in wines are ochratoxin B (OTB), ochratoxin C (OTC), OTα, and ochratoxin β (OTβ). Other forms identified are ochratoxin A methyl ester (MeOTA), ochratoxin B methyl ester (MeOTB), ochratoxin B ethyl ester (EtOTB), OTα methyl ester, OTA ethyl amide, and OTA glucose ester [8,10,93].

Freire et al. [10] reported that the fungus could produce these mycotoxins, due to the activity of the yeast utilized for fermentation or by reacting with grape and must constituents. Certain *Aspergillus* strains have been shown to be capable of converting OTA into its metabolites through e processes of hydrolysis and conjugation. Concerning OTα formation, for example, it is plausible to speculate that *A. carbonarius* uses the phenylalanine contained in the parental mycotoxin as a source of nitrogen [94]. In addition, Freire et al. [1] stated that yeasts can also form modified mycotoxins during the alcoholic fermentation. *S. cerevisiae*, for example, was reported to produce extracellular enzymes, in particular glucosidase, pectinase, and xylanase, that can affect the hydrolysis of OTA or its bond with other components of the must during the fermentation process, thus forming various derivatives [10]. Finally, the formation of OTA derivatives is also linked to a matrix association phenomenon. In this case, the pH of the must favors carboxylic acid transformation to an ethanolic ester in OTA, ionization of the amino group of the parental mycotoxin, or esterification reactions [8]. There can also be ionic bonds between OTA and some components of grapes, such as polysaccharides, pectic substances, lignin, and proteins [10].

In 1996 the first account of the simultaneous appearance of OTA and OTC in wines was reported; it was determinated that OTC constituted about 10% of the total OTA amount [22]. Furthermore, Remiro et al. [95] found detectable levels of OTA and OTB in all the 51 red wines from Navarra (Spain) analyzed; 71% contained OTC, and 18% presented OTA, OTB, MeOTA, MeOTB, OTC, and EtOTB simultaneously. In another study, Remiro et al. [23] proved the co-occurrence of OTA, OTB, OTC, MeOTA, MeOTB, and EtOTB in 96 red Mediterranean wines. 

An aspect that should be kept in mind while studying modified mycotoxins is their effect on human and animal health. Ortiz-Villeda et al. [8] reported that OTA is the ochratoxin with the highest toxicity but its derivates in co-mixtures with other micotoxins could also potentially be a grave danger to human health. Indeed, these forms might have synergistic and/or additive health effects [10]. Furthermore, both human and animal metabolism can turn modified ochratoxins into OTA, improving its bioavailability, which is another great cause of concern [96]. Confirming this, Remiro et al. [95] found that OTC was reconverted to the parental mycotoxin in red wine; thus, through the exclusive use of OTA detection, the total ochratoxin intake may be undervalued. Ortiz-Villeda et al. [8] summarized available knowledge saying that the presence of these forms in wine may develop into different cases. The first one is a reduction or total removal of the starting toxicity; the second one is that these compounds may possess the same toxicity as the original molecule or possibly even more; the third one is the synergism with the parental mycotoxin or other wine compounds; the last one is that these forms can be reconverted into OTA.

The evaluation of these molecules is a second issue correlated with their possible presence in wine. Indeed, since these forms have a different structure than their native form, a traditional analysis may not result in their detection. Underestimating these derivatives could result in a dangerous scenario for public health. Thus, it is crucial to search for sensitive and selective analytical methods for their identification.

## 5. Conclusions

This paper focuses on OTA’s fate during the vinification process, considering the most recent studies on the topic. Generally, almost all the studies agree that, during winemaking, there is a reduction in OTA, and only during maceration is an increase in OTA content observable. This is why red wines generally contain higher OTA levels than white ones, focusing on the vinification process. Instead, in all the other steps of vinification, including alcoholic and malolactic fermentation, the OTA contamination almost always decreases, with a wide range of values that are strictly related to the different conditions in which the process takes place. Furthermore, some fining agents, such as gelatin, egg albumin, bentonite, and AC, further reduce OTA levels. Depending on the compound used, a wide variation is reported in OTA reduction, the most effective having the strongest impact on wine color. This is another step contributing to white wine being less contaminated.

## Figures and Tables

**Table 1:** OTA removal with chemical methods.

Fining Agent	Characteristics of Fining Agent	Dosage (g/hL)	Wine Color	OTA Reduction (%)	References
Gelatin		10 mL/hL	Red	58	[51]
	AlCl_3_ activated	100	Red	17–30	[81]
	High molecular weight in sheets	16	Red	39	[80]
		10	Red	14	[62]
		10 mL/hL	Red	8–21	[52]
		20	Red	80	[82]
	Gelatin with a high degree of hydrolysis	15	Red	0	[73]
	Gelatin with a low degree of hydrolysis	10	Red	3	[73]
Egg albumin		16	Red	16	[80]
		10	Red	20	[62]
		10	Red	22–34	[52]
		20	Red	94	[82]
		15	Red	9	[73]
Isinglass		10	Red	21	[62]
Bentonite		80	Red	34–40	[83]
		50	Red	7–17	[52]
		250	White	67	[59]
		50	White	20	[62]
	Activated sodium	10–60	Red	22–33	[80]
		100	Red	8	[81]
		10–20	Red	1–12	[42]
		100	White	0–6	[45]
		10–20	Red	10	[82]
		75	Red	17	[73]
Activated Carbon (AC)		20–100	White	13–98	[45]
		25	White	17	[62]
		10–100	Red	36–90	[42]
	Decolorizing	30	Red	73	[34]
	KOH activation	20	Red	54	[84]
		100	White	100	[85]
Gelatin–bentonite		7 + 50	White	20	[62]
Casein–gelatin–bentonite		5 + 7 + 50	White	21	[62]
Polivinilpolipirrolidone (PVPP), plant protein and amorphous silica		10–50	Red	30–40	[80]
Chitin		500	Red	29	[80]
		500	Red	67	[86]
		500	White	53	[86]
Chitosan		500	Red	67	[80]
		500	Red	83	[86]
		500	White	53	[86]
		1000	Red	100	[87]
Oakwood	Powder	800	Red	76	[88]
	Chips	800	Red	50	[88]
PA-EGDMA ^1^		500	Red	68	[89]
Kaolinite		100	Red	<1	[81]
Celite		100	Red	<1	[81]
KFeCN ^2^		10	Red	7–8	[81]
Silica gel	AlCl_3_ activated	100	Red	18–34	[81]
	OH	100	Red	1–11	[81]
		50	Red	18	[34]
Zeolite		100	Red	2–5	[81]
Cellulose	Microcrystalline	100	Red	<1	[81]
	Short fiber	50	Red	16	[81]
Carrageenan		100	Red	5–9	[81]
Pectin		100	Red	3–9	[81]
Potassium-caseinate		100	Red	24–35	[81]
		75	Red	11	[73]
		10–250	White	5–15	[59]
PVPP		16	White	0	[52]
		20–80	White	10–12	[59]
		50	Red	14	[34]
		250–500	Red	14–53	[89]
PVP-DEGMA-TAIC ^3^		250–500	Red	51–70	[89]
Casein		5	White	8	[62]
		40	White	0–13	[52]
Vegetable protein from peas		20	Red	11	[73]
Cholestyramine		10–100	Red	6–35	[59]
KSF-montmorillonite		5–1000	Red	52–100	[87]

^1^ poly(acrylamide-co-ethylene glycol-dimethacrylate). ^2^ potassium-ferrocyanide. ^3^ resins of copolymerization of N-vinyl-2-pyrrolidinone with ethylene glycol dimethacrylate and triallyl isocyanurate.

## Data Availability

No new data were created or analyzed in this study. Data sharing is not applicable to this article.
